# Myoinositol Improves Embryo Development in PCOS Patients Undergoing ICSI

**DOI:** 10.1155/2016/6273298

**Published:** 2016-09-29

**Authors:** Artur Wdowiak

**Affiliations:** Diagnostic Techniques Unit, Department of Health Sciences, Medical University of Lublin, Lublin, Poland

## Abstract

The aim of this study was to investigate the activity of myoinositol, in a court of 217 PCOS women undergoing intracytoplasmic sperm injection (ICSI), on pregnancy rate, embryo development, estradiol, and progesterone concentration in blood serum, superoxide dismutase (SOD), and catalase (CAT) in follicular fluid. Concerning the court of patient, 112 (groups I and II) out of 217 were PCOS women, whereas group III consisted of healthy subjects (not PCOS). Group I patients were treated with 400 *μ*g of folic acid per day for 3 months before ICSI, whereas group II patients received 4000 mg of myoinositol and 400 *μ*g of folic acid per day for 3 months before ICSI. Group II revealed a shorter embryo/blastocyst development period between microinjection and 5-cell stage compared to group I. The difference in SOD concentration between groups I and II and between groups II and III was statistically significant. In group II, 34.62% of pregnancies were obtained, whereas in group I this number reached 20% (NS). Myoinositol increased embryo development dynamics and accelerated blastocyst stage reaching time; however, no effect was shown on clinical pregnancy. Furthermore, it restored SOD concentration, lowered in PCOS women, but did not exert any effect on CAT concentration.

## 1. Introduction

Polycystic ovary syndrome (PCOS) is the most commonly reported ovulation disorder in women [1–3]. It is characterized by considerable heterogeneity in both clinical and hormonal signs [4]. In certain cases, the in vitro fertilization (IVF) is the main treatment for patients with this particular diagnosis.

Success of IVF depends on multiple factors [5] and especially on obtaining a good quality of embryos. Oestradiol and progesterone are two crucial hormones engaged in ovum development. Proper concentrations of these hormones in blood serum ensure a reproductive success in assisted reproductive technology (ART) [6].

An antral follicle constitutes a microenvironment, where ovum develops, which simultaneously influences the future quality of embryo. Oxidative stress plays an important role during folliculogenesis and oogenesis, although our knowledge related to its effects remains still insufficient [7]. It is also well known that PCOS is often associated with certain disorders within the redox system, which depend on the activity of superoxide dismutase (SOD) and catalase (CAT) enzymes, next to other factors [8].

As far as PCOS is concerned, it was possible to confirm several degrees of insulin resistance reported in patients, which could also influence fertility treatment success [1–3, 9, 10]. Myoinositol (myo-Ins), as a mediator of the insulin activity, is considered today a therapeutic agent commonly administered in IVF for the ovulation induction [11–14]. Since myo-Ins is a secondary transmitter of intracellular activity of folliculotropic hormone (FSH), it was assumed that PCOS patients have a deficiency of this hormone, which leads to a disturbance of FSH signalling, causing worsened quality of oocytes, as highlighted in a seminal review, recently published [15]. Therefore, myo-Ins deficiency may also affect the dynamics of embryonic development, as well as the IVF procedure efficiency in patients with PCOS [15].

The primary outcome of this study was to evaluate the influence of myo-Ins on the pregnancy rate and on the dynamics of embryo development in PCOS patients undergoing ICSI. As secondary outcome, our study aimed at testing the myo-Ins effects on oestradiol and progesterone concentration in blood serum, as well as on SOD and CAT concentration in the follicular fluid of these patients. We considered not only the well-known mechanisms of action, proper to myo-Ins, based on the improvement of insulin and FSH signalling, but also their possible activity as antioxidant molecule.

## 2. Materials and Methods

This retrospective study was carried out on the data of patients undergoing ART in 2013 and 2014 at the Ovum Fertility Treatment Centre in Lublin. The study covered 217 women treated for infertility by means of intracytoplasmic sperm microinjection (ICSI) technique. All patients were qualified to undergo ICSI due to a moderate, masculine infertility factor that makes it impossible to conduct classic IVF. A total of 198 from treated pairs had 4 to 6 previous intrauterine inseminations during the last 1-2 years that did not lead to pregnancy, and in the case of 19 women we have reported bilaterally blocked fallopian tube. For the subjects enrolled into the PCOS groups, the inclusion criteria required that such patients had to meet the Rotterdam criteria (2004). Women constituting the studied group were between 27 and 35 years old; they all had FSH < 10 IU/mL and appropriate AMH (Anti-Müllerian hormone) value. The exclusion criteria were the following: presence of severe endometriosis, BMI < 17 and >30, and metabolic diseases as well as lowered ovarian reserve.

The study obtained a consent of Bioethical Committee at the Institute of Rural Health in Lublin. All patients signed an informed consent before participating to the study.

All patients were treated with the ICSI procedure, taking advantage of fresh oocytes and fresh sperm.

GnRh analogues (Diphereline: Ipsen Pharma) and FSH recombinants (Gonal-F: Merck-Serono, Puregon: Organon) were used in short protocols to stimulate ovulation from the 3rd cycle day (to a maximum of the 17th cycle day). In the day of implementing ovulation induction, the oestradiol (E_2_) (pg/mL) and progesterone (ng/mL) levels were detected in the morning. These two parameters were determined when the largest oocyte in the evaluation of ultrasound exceeded 17 mm diameter. The puncture was conducted 36 hours after administering recombinant HCG (r-hCG) (Ovitrelle: Merc-Serono). A total of 52 patients (group II) among the PCOS subjects were administered with 4000 mg of myo-Ins and 400 *μ*g of folic acid (folate) (Inofolic: Temapharm, Poland) for 3 months before undergoing ICSI. The other PCOS subjects were treated with 400 *μ*g of folic acid alone.

Follicular fluid was collected from follicles with diameter exceeding 17 mm. When the follicular fluid did not contain ovum or was contaminated with blood, the sample was excluded from the study. SOD and CAT were determined on puncture day or on collection day of oocytes, which means between the 11th and the 19th cycle day. SOD activity was measured spectrophotometrically using SOD Assay Kit (Sigma-Aldrich), whereas CATs were detected with the Catalase Assay Kit (Sigma-Aldrich) according to the manufacturer instructions. The final level of the SOD activity and CAT activity was reported as a unit of enzymatic activity per protein mg (mIU/mg).

Oocytes were separated from cells in the granular layer, which was followed by the ICSI procedure 3 hours after ovarian puncture. The inseminated cells were grown in 25 *μ*L drops of Cleavage medium (COOK, Sydney IVF, Australia) under mineral oil in an automatic 5% CO_2_ incubator at 37°C until the second day (stage of 2–5 cells). Fifty hours after the ICSI, the culture medium was changed with blastocyst medium (COOK, Sydney IVF, Australia).

Embryo culture was evaluated by means of constant monitoring performed in 10-minute intervals with a camera placed inside the incubator. During all the observation period, the embryos remained in the incubator. The *t*_0_, *t*_*F*_, and *t*_*C*_ times were defined as the hour of the ICSI, the first moment when pronuclei became visible, and the last moment of their visibility, respectively. The moment when a single cell embryo appeared after syngamy was defined as *t*_1_, and then the superseding divisions as *t*_2_, *t*_3_, *t*_4_, *t*_5_, *t*_6_, *t*_7_, and *t*_8_. The beginning of morula formation was called *t*_*M*_, whereas *t*_*B*_ is when the first signs of blastocyst cavity could be seen. Blastocysts were evaluated according to criteria declared by ASRM and ESHRE, and only one of them was transferred in order to avoid multiple pregnancy. During the 7th week of pregnancy, the echo of the embryo and heart rate were evaluated by means of ultrasound examination.

## 3. Statistical Analysis

The measurable parameters were shown as mean and standard deviation, whereas the nonmeasurable ones were presented as numerical amount and percentage.

For qualitative features, a Chi^2^ test was utilized to detect any existing differences. The ANOVA variance analysis was used to test differences among groups, whereas R Pearson's correlation was used to verify dependence between embryo development times and selected parameters. The difference was considered statistically significant at *p* < 0.05. The database and statistical tests were conducted using Statistica 9.1 programme (StatSoft, Poland).

## 4. Results

**Figure 1:**
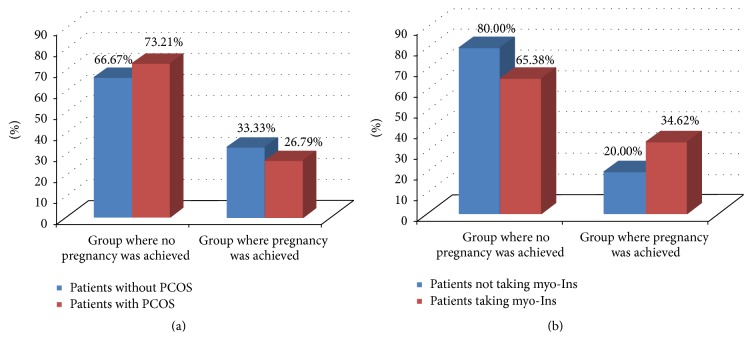
Pregnancy percentage. (a) Percentage layout of pregnancies in the group with PCOS and without PCOS. Chi^2^ = 1.107, df = 1, and *p* = 0.293; (b) percentage layout of pregnancies in the group with PCOS among patients taking myoinositol and patients who were not taking this preparation (Chi^2^ = 3.034, df = 1, *p* = 0.081).

Patients were divided into the following groups: group with PCOS women (*n* = 112) and group with healthy patients (*n* = 105). In the first group, myo-Ins was administered to 52 women (treated group), whereas 60 were treated with 400 *μ*g of folic acid alone (control group). Both treatments were daily and lasted for 3 months before ICSI. Among the healthy subjects (healthy group), we obtained 33.33% of pregnancies, whereas among the PCOS women this value reached 26.79%. These differences were not statistically significant (Chi^2^ = 1.107, df = 1, and *p* = 0.293) (Figure 1(a)). However, PCOS patients, under myo-Ins administration, achieved 34.62% pregnancies, whereas in controls just 20% of pregnancies were recorded. These differences were statistically insignificant (Chi^2^ = 3.034, df = 1, and *p* = 0.0810) (Figure 1(b)).

When we compared the embryo development times between the groups of PCOS controls (group I), PCOS patients treated with myo-Ins (group II), and healthy subjects (group III), statistically significant differences were observed: *t*_*F*_ (*F* = 4.743, *p* = 0.010) between group I and group III and *t*_*C*_ (*F* = 14.724, *p* < 0.001) between group I and group II, as well as groups I and III. Of note, statistically significant differences were observed between group I and group II, as well as group I and group III, in the following times: *t*_1_ (*F* = 8.388, *p* < 0.001), *t*_2_ (*F* = 17.287, *p* < 0.001), *t*_3_ (*F* = 9.762, *p* < 0.001), *t*_4_ (*F* = 18.135, *p* < 0.001), *t*_5_ (*F* = 9.123, *p* < 0.001), and finally *t*_*B*_ (*F* = 19.326, *p* < 0.001), as shown in Table 1. In the remaining times, no statistically significant differences were observed.

**Figure 2:**
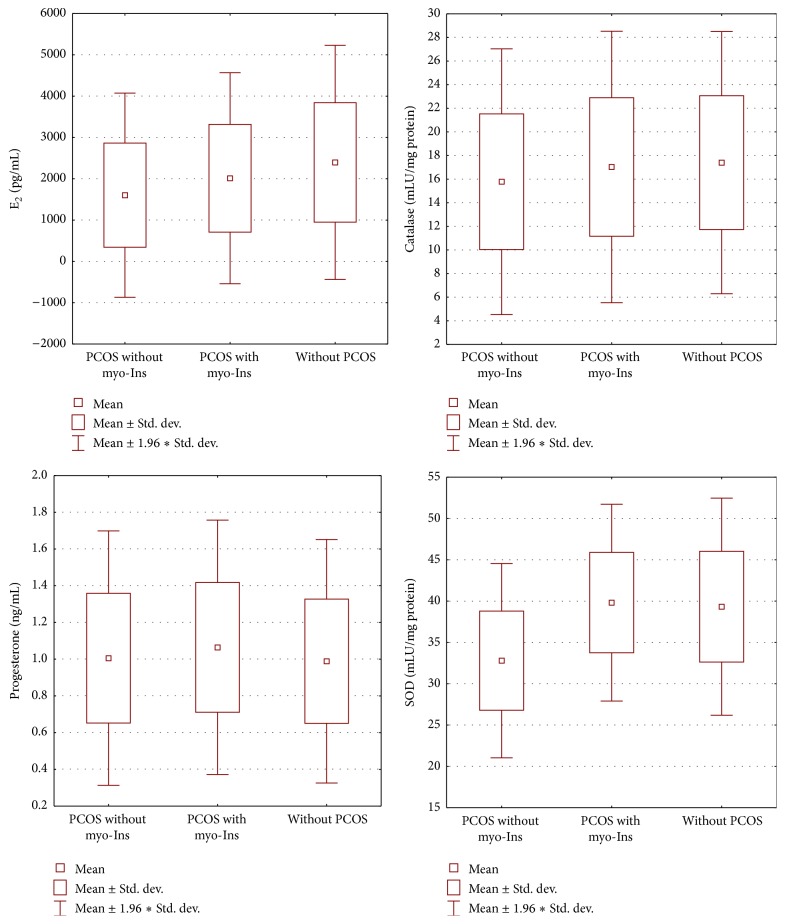
Parameters levels. Differences between oestradiol, progesterone, SOD, and CAT levels in groups with PCOS not taking myoinositol (group I) and taking myoinositol (group II) and in group without PCOS (group III).

The values of oestradiol in the day of ovulation induction significantly differed between group I and group III (*F* = 6.558, *p* = 0.002), whereas in the same day progesterone did not show significant differences. The concentration of SOD in follicular fluid revealed statistically significant differences among the three groups, namely, between group I and group II as well as between group II and group III (*F* = 24.051, *p* < 0.001). On the contrary, no significant differences were observed for CAT in follicular fluid (Figure 2).

The influence of oestradiol, detected in serum, on the embryo development times showed negative correlations between its level in group I and *t*_*F*_ (*r* = −0.307, *p* = 0.017), *t*_4_ (*r* = −0.321, *p* = 0.013), *t*_5_ (*r* = −0.316, *p* = 0.014), and *t*_*B*_ (*r* = −0.312, *p* = 0.015). In the above-mentioned times, the increase in oestradiol level exerted a considerable influence on embryo developmental dynamics; however, no effect was observed in the remaining times. In group II, similar negative correlations concerned E_2_ and *t*_1_ (*r* = −0.358, *p* = 0.009), *t*_3_ (*r* = −0.294, *p* = 0.035), *t*_4_ (*r* = −0.283, *p* = 0.042), and *t*_5_ (*r* = −0.365, *p* = 0.008). In the other times no such statistically significant correlations were detected. Group III showed negative correlations only between E_2_ and the following times: *t*_4_ (*r* = −0.247, *p* = 0.011), *t*_6_ (*r* = −0.221, *p* = 0.023), and *t*_*B*_ (*r* = −0.192, *p* = 0.049). In group I a positive correlation between progesterone level and *t*_2_ (*r* = 0.284, *p* = 0.028) was found, whereas in group II negative correlations between progesterone level and *t*_*F*_ (*z* = −0.303, *p* = 0.029) and *t*_5_ (*z* = −0.309, *p* = 0.026) were reported, and in group III a negative correlation between progesterone level and *t*_8_ (*z* = −0.196, *p* = 0.045) was reported, next to a positive correlation with *t*_*B*_ (*z* = 0.253, *p* = 0.009). No other statistically significant correlations concerning progesterone level appeared. In PCOS patients treated with myo-Ins (group II), researchers observed a negative correlation between SOD levels in follicular fluid and the following times: *t*_*F*_ (*r* = −0.318, *p* = 0.013), *t*_*C*_ (*r* = −0.273, *p* = 0.035), *t*_1_ (*r* = −0.484, *p* = 0.000), *t*_2_ (*r* = −0.402, *p* = 0.001), *t*_3_ (*r* = −0.332, *p* = 0.010), *t*_4_ (*r* = −0.400, *p* = 0.002), *t*_5_ (*r* = −0.408, *p* = 0.001), *t*_9_ (*r* = −0.285, *p* = 0.027), and *t*_*B*_ (*r* = −0.341, *p* = 0.008). In the other times, no significant dependencies were observed. Moreover, in these subjects no significant correlations between SOD and embryo developmental times were discovered. In healthy patients a negative correlation was observed for the same parameter only with *t*_*M*_ (*r* = −0.335, *p* = 0.000). In group I positive correlations were observed between CAT levels and *t*_5_ (*r* = 0.338, *p* = 0.008) as well as *t*_*B*_ (*r* = 0.279, *p* = 0.031), and nothing else. In group II we can see positive correlations between the activity of CAT and *t*_*F*_ (*r* = 0.335, *p* = 0.015) and *t*_1_ (*r* = 0.293, *p* = 0.035), next to *t*_5_ (*r* = 0.627, *p* < 0.001). In group III positive correlations between CAT concentration and *t*_1_ (*r* = 0.376, *p* < 0.001), *t*_2_ (*r* = 0.4, *p* < 0.001), *t*_3_ (*r* = 0.197, *p* = 0.044), and *t*_4_ (*r* = 0.274, *p* = 0.005) were noted, without other significant correlations (Table 2).

## 5. Discussion

Our study proved that myo-Ins-based therapy, beside its well-known insulin-lowering action, increases the dynamics of embryo development, as well as the activity of SOD in follicular fluid. On the other hand, myo-Ins did not modify CAT activity in follicular fluid. It is important to highlight that SOD activity maintains the balance of redox system, by eliminating superoxide anions and creating H_2_O_2_. This is the first line of protection against free radicals [8, 16]. The effects on SOD are in perfect agreement with the antioxidant activity which can be ascribed to myo-Ins. Although there is not experimental evidence obtained in mammals, some interesting results coming from researches in fishes have demonstrated that myo-Ins administration can enhance antioxidant defences. In such way, it reduces lipid peroxidation and protein oxidative damage. Various data have shown that myo-Ins opposes detrimental ROS activity and enhances immunity. This molecule looks to activate Nrf2 signalling which plays a key role in inducing gene transcription of antioxidant enzymes and therefore in keeping the physiological redox status [17]. It is a dynamic balance, essential to maintain a healthy condition, and its breaking leads to several pathologies and tissue damage. Among these harmful effects, the abnormalities of redox system stand as the main cause underlying the alterations of genetic material in the ovum [7, 18]. Only the ovum is capable of repairing DNA damage in the spermatozoon; however, this depends on the severity and type of irregularity, as well as the amount of oocytes [19]. It can be therefore expected that myo-Ins treatment may protect against adverse epigenetic changes, and effects of such changes can be evaluated only by examining the condition of a child conceived with the ICSI method [20, 21].

Embryo development times that we were able to obtain during our measurements can be compared with the times that Azzarello et al. and Kirkegaard et al. described in literature [22, 23].

In studies conducted by Gerli et al. the researchers were able to compare a group of 45 women, treated with myo-Ins and folic acid, with 47 women treated only with folic acid. myo-Ins group revealed significantly higher oestradiol levels when compared with the untreated group [13].

Papaleo et al. compared protocols concerning ICSI stimulation with rFSH (recombinant FSH) and Myo-ins with 30 rFSH obtained from 30 patients and reported a lower oestradiol concentration in myo-Ins group, which is not compliant with results obtained by Gerli et al. and the results obtained in our study [12, 13]. They also observed a higher percentage of pregnancies per cycle in myo-Ins group: 33.3% when compared to 13.3% in the group without myo-Ins. In our studies the percentage of pregnancies in the group with myo-Ins was almost identical (34.62%) to the one obtained by Papaleo et al., whereas it reached 20% in the control group.

Chiu et al. conducted studies on the group of 53 female patients taking myo-Ins during stimulation before undergoing IVF and they observed a lowered level of myo-Ins in follicular fluid collected from samples with immature oocytes [11]. Their studies indirectly confirm our results, as far as the influence exerted by myo-Ins on the dynamics of embryo development is concerned.

Seleem et al. compared SOD concentration in follicular fluid of 20 PCOS patients and in a group of 20 healthy patients during the ICSI procedure, and they reported statistically significant higher SOD levels in the group without PCOS, in accordance with our results [24].

In the studies conducted by Muñoz et al., 774 IVF cycles were analysed by means of the technology based on observing embryos in real time, as we made in our study [25]. Authors reported the existence of a significant influence exerted by oestradiol levels on embryo development. Similarly, to our studies, they observed a considerable influence of high E_2_ values on increasing the embryo development times; however, they used a different method of statistical analysis and a diverse study model (they divided patients into four groups, depending on the E_2_ value in serum, and then they compared the times of embryo development between particular groups) to analyse their results. In this way, it was impossible to unambiguously relate their results. Similarly, to our findings, Muñoz et al. reported that progesterone concentration in blood serum exerts an influence on the dynamics of embryo development only to a slight extent.

Studies devoted to fertility treatment in PCOS patients require further tests, using more numerous groups of patients and with more standardised diagnostic techniques to understand in-depth some specific points; however, the studies so far performed have confirmed the validity of using myo-Ins in ART with PCOS patients.

## 6. Conclusions

The use of myo-Ins before and during IVF stimulation increases the dynamics of embryo development in the first two days of culture and reduces the amount of time required to achieve the blastocyst stage. However, the influence of these results in the number of clinical pregnancy was not confirmed.

The concentration of superoxide dismutase in follicular fluid is lower in PCOS women when compared with healthy patients; however, when PCOS women are treated with myo-Ins, this concentration reaches values that are observed among healthy women. The effect of oestradiol concentration in serum, as well as the activity of SOD in follicular fluid, increases the dynamics of embryo development. On the other hand, myo-Ins did not have any influence on CAT activity in follicular fluid. SOD increase may be explained by means of the antioxidant activity exerted by myo-Ins, whereas its inefficacy on the rise of CAT levels should be investigated in-depth. The results we obtained confirm also the necessity to conduct further studies focusing on myo-Ins therapy.

## Figures and Tables

**Table 1:** Embryo development times.

	PCOS patients treated with folic acid (group I)	PCOS patients treated with myoinositol plus folic acid (group II)	Patients without PCOS (group III)	*p*	Difference between groups
*t*_*F*_	10.38 ± 3.08	9.57 ± 3.24	8.86 ± 2.96	0.010	I-III
*t*_*C*_	26.14 ± 3.29	22.53 ± 3.98	23.83 ± 3.60	<0.001	I-II, I-III
*t*_1_	26.03 ± 3.30	23.46 ± 3.61	24.23 ± 3.54	<0.001	I-II, I-III
*t*_2_	29.14 ± 3.63	26.60 ± 3.73	25.86 ± 3.25	<0.001	I-II, I-III
*t*_3_	39.43 ± 4.74	35.53 ± 5.83	36.58 ± 4.61	<0.001	I-II, I-III
*t*_4_	42.85 ± 6.69	37.04 ± 4.02	38.45 ± 5.38	<0.001	I-II, I-III
*t*_5_	51.84 ± 7.98	48.16 ± 8.10	53.72 ± 7.28	<0.001	I-II, I-III
*t*_6_	54.03 ± 7.02	53.48 ± 7.41	55.85 ± 8.11	0.130	
*t*_7_	58.05 ± 9.43	57.64 ± 8.51	57.86 ± 8.88	0.972	
*t*_8_	60.50 ± 11.43	63.17 ± 13.36	62.58 ± 12.54	0.468	
*t*_9_	76.82 ± 11.03	74.72 ± 12.22	76.19 ± 11.65	0.619	
*t*_*M*_	83.79 ± 11.25	85.70 ± 11.23	85.57 ± 11.94	0.584	
*t*_*B*_	107.56 ± 4.40	104.59 ± 1.66	104.92 ± 2.32	<0.001	I-II, I-III

Differences between embryo development times in groups without PCOS and groups with PCOS, both taking and not taking myoinositol.

**Table 2:** Embryo development times and parameters levels.

		PCOS patients treated with folic acid (group I)				PCOS patients treated with myoinositol plus folic acid (group II)				Patients without PCOS (group III)			
		E_2_	Progesterone on the day of ovulation induction	SOD	Catalase	E_2_	Progesterone on the day of ovulation induction	SOD	Catalase	E_2_	Progesterone on the day of ovulation induction	SOD	Catalase
*t*_1_	*r*	−0.164	0.038	−0.484	0.082	−0.358	0.128	−0.036	0.293	−0.055	−0.055	−0.001	0.376
	*p*	0.210	0.775	<0.001	0.533	0.009	0.366	0.801	0.035	0.579	0.581	0.993	<0.001

*t*_2_	*r*	−0.130	0.284	0.402	0.036	−0.146	0.273	−0.092	0.190	−0.057	0.085	−0.093	0.400
	*p*	0.323	0.028	0.001	0.787	0.303	0.050	0.515	0.178	0.561	0.390	0.343	<0.001

*t*_5_	*r*	−0.316	−0.122	−0.408	0.338	−0.365	−0.309	0.154	0.627	−0.084	0.137	−0.016	0.159
	*p*	0.014	0.352	0.001	0.008	0.008	0.026	0.276	<0.001	0.392	0.163	0.868	0.106

*t*_*M*_	*r*	0.028	−0.041	−0.053	−0.014	0.234	0.159	0.120	0.043	−0.136	0.086	−0.335	−0.137
	*p*	0.831	0.758	0.689	0.915	0.096	0.260	0.396	0.765	0.168	0.382	<0.001	0.162

This table shows only the significant correlations between embryo development times and the level of oestradiol and progesterone in serum and SOD and CAT in follicular fluid.
